# A Case of Pneumocephalus Due to an Untreated Atrial-Esophageal Fistula Post-Left Atrial Ablation Therapy

**DOI:** 10.7759/cureus.73538

**Published:** 2024-11-12

**Authors:** Emman Fatima, Ian Hill, Sahar Fatima, Mehmet H Akay, Iqbal Ratnani

**Affiliations:** 1 Critical Care Medicine, Houston Methodist Hospital, Houston, USA; 2 Critical Care Medicine, Creighton University School of Medicine, Omaha, USA; 3 Cardiothoracic Surgery, Houston Methodist Hospital, Houston, USA

**Keywords:** atrial fibrillation management, critical cardiovascular incidents, left atrial ablation, left atrial ablation atrioesophageal fistula, neurological complication

## Abstract

Air embolisms can be caused by trauma, barotrauma, or due to surgical procedures in neurosurgery, vascular surgery, and cardiac surgery. An atrial-esophageal fistula (AEF) is a life-threatening complication that can occur following left atrial ablation therapy, which is used to treat refractory atrial fibrillation (Afib). AEF, if left untreated, can lead to serious neurological complications such as pneumocephalus. We present a rare case of pneumocephalus in a 60-year-old male who recently underwent left atrial ablation therapy after which he presented to the ER following a fall. On examination, the patient was confused and had difficulty getting up. A left hemiparesis and a rightward gaze preference were also observed. The CT scan of the head confirmed pneumocephalus. Our report is unique as it explores a very rare entity and highlights the implications of a delay in the treatment of AEF. Early surgical intervention is the key to ensuring the survival of patients with an AEF.

## Introduction

An atrial-esophageal fistula (AEF) is defined as an aberrant communication channel between the left atrial wall and the esophagus. This often occurs as a late complication of severe thermal injuries to the esophagus and surrounding structures after left atrial ablation therapy [1]. Air embolisms result from untreated AEF and can often manifest as neurologic and cardiac complications. Altered mental status, meningitis, seizures, strokes, sepsis, transient ischemic attacks (TIAs), coma, and cardiac arrest can arise due to air emboli entering the systemic circulation [1-2]. Patients with these complications present with stroke-like symptoms, and are more likely to be admitted to stroke units or infectious disease wards for evaluation [3]. As AEF is rare, its diagnosis can be difficult, and no predictors of lesion development have been established. Current preventative strategies involve reducing the power during pulmonary vein isolation (PVI) or when the posterior left atrial wall is being ablated, to limit the duration and energy frequency delivered to avoid overlapping ablation lines [3].

AEF can often lead to pneumocephalus. Pneumocephalus, also known as intracranial aerocele or pneumatocele, refers to the presence of air in the intracranial region [4]. Symptoms of pneumocephalus vary but the condition is most often associated with confusion, nausea, dizziness, headache, seizures, and focal neurological symptoms such as hemiparesis or cranial nerve palsy. While pneumocephalus is generally harmless, if it is accompanied by elevated intracranial pressure, it can lead to tension pneumocephalus, which is fatal [5,6]. We report a case of pneumocephalus resulting from AEF caused by left atrial ablation therapy.

## Case presentation

**Figure 1:**
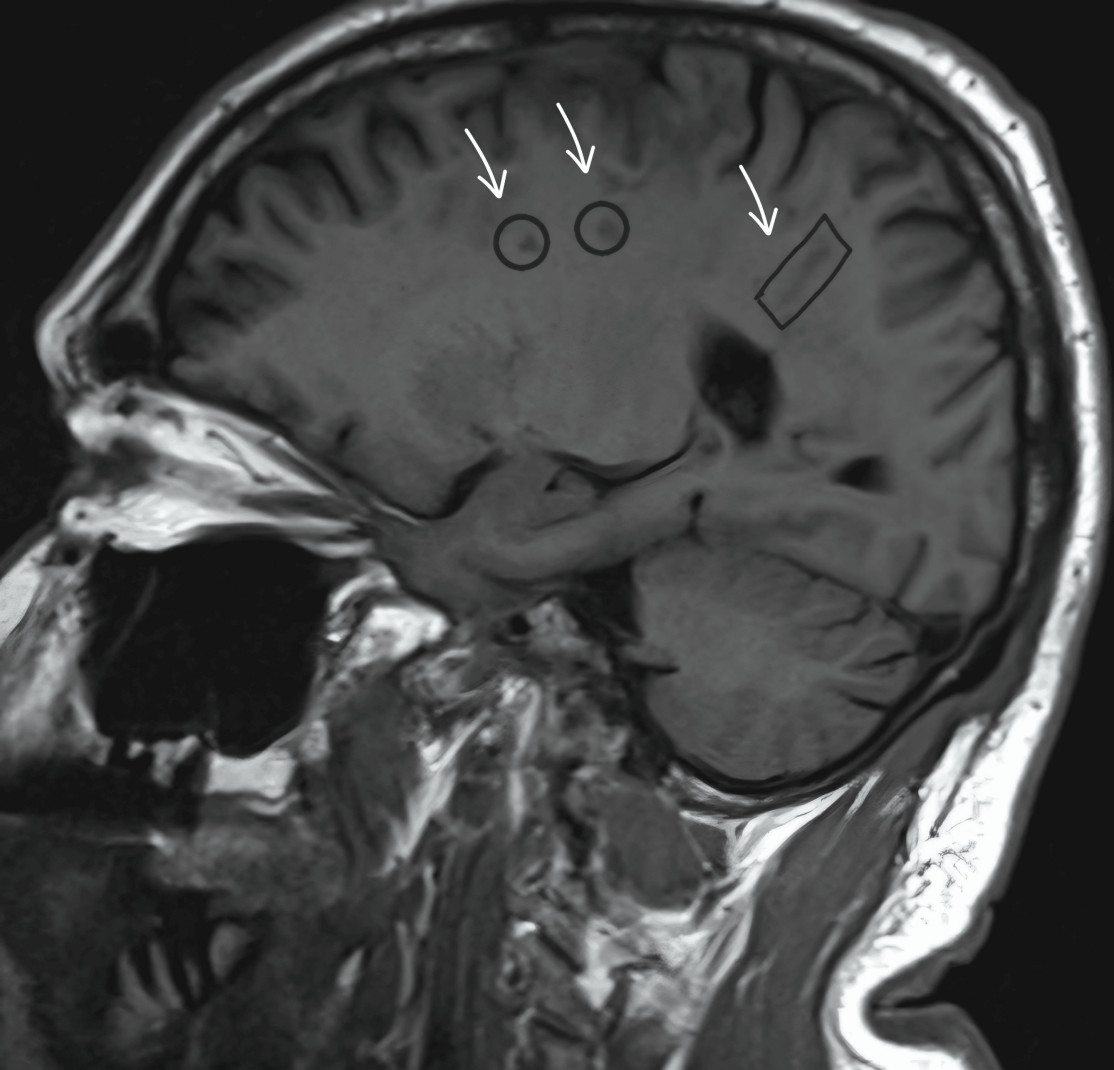
MRI brain - air pockets encircled and marked by white arrows MRI: magnetic resonance imaging

**Figure 2:**
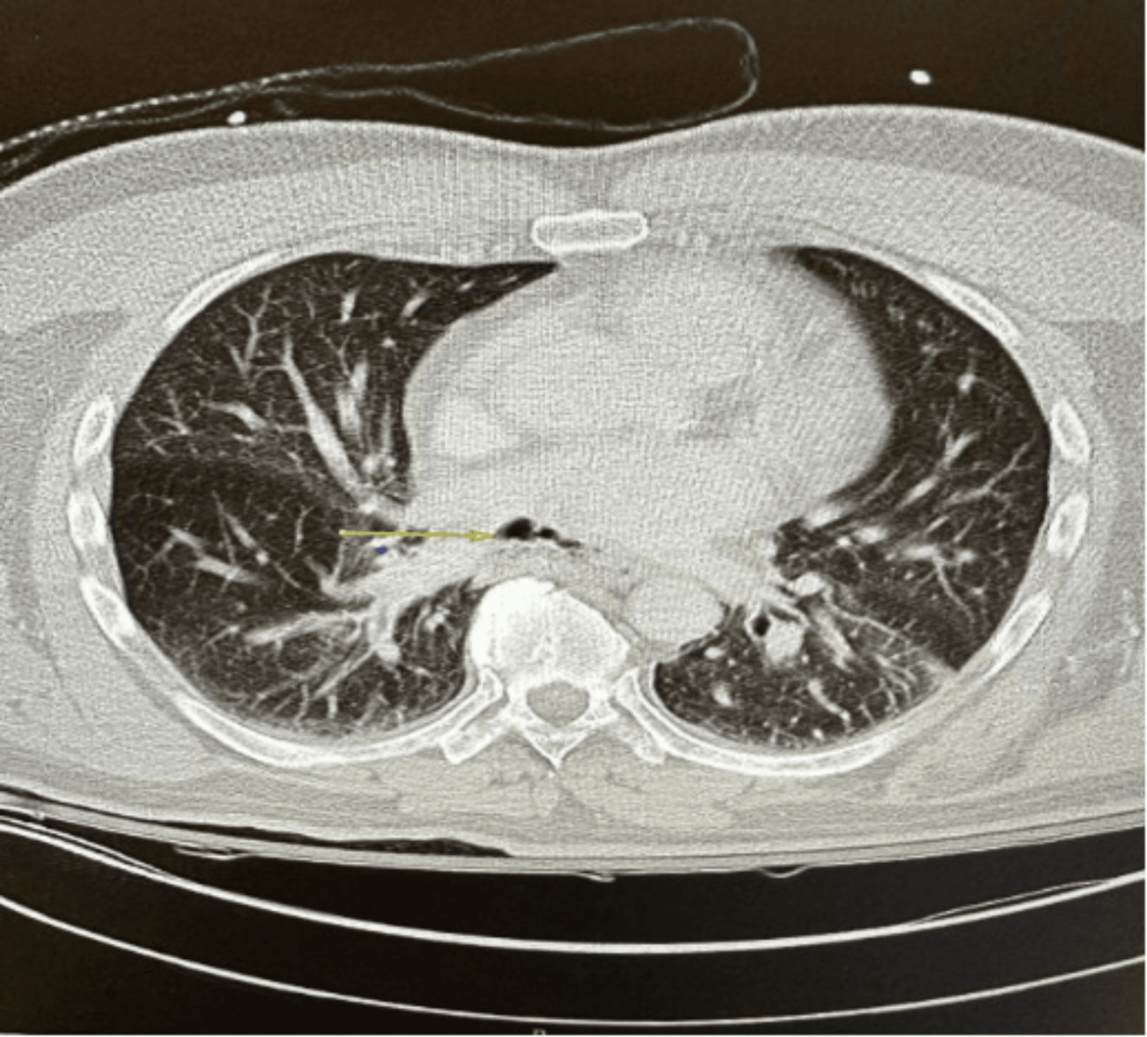
Axial thoracic CT scan: atrial-esophageal fistula marked by yellow arrow CT: computed tomography

A 60-year-old male with a past medical history of hypertension, asthma, and atrial fibrillation (Afib) presented to the Emergency Room after a fall. His past surgical history was significant for pulmonary vein ablation. On examination, he had a blood pressure of 166/88 mmHg, temperature of 98.4 °F, heart rate (HR) of 109 beats per minute, respiratory rate (RR) of 22 breaths per minute, and SpO_2_ 100%. He was confused and demonstrated left-sided weakness and a rightward gaze preference. Differential diagnoses included transient ischemic attack (TIA) and stroke. A CT scan and MRI brain revealed air pockets within the parietal lobes and pneumocephalus was confirmed. CT scan of the chest revealed an AEF and a small posterior pneumomediastinum (Figures 1, 2).

This finding led to the diagnosis of AEF resulting in pneumatocele, which prompted emergency surgical intervention. Emergent open surgical repair of the left atrial and esophageal wall was performed. Intraoperative findings included a defect between the right pulmonary vein and the posterior left atrial wall. Necrosis between the left atrium and the esophagus was also observed. Postoperatively, the patient’s recovery was impacted by Lactobacillus-associated mediastinitis, severe sepsis, and several splenic and cerebral embolic infarcts. His condition was further complicated by recurrent refractory Afib, gastrointestinal bleeding, and anticoagulation management difficulties. Despite extensive medical and surgical interventions, the patient did not survive and succumbed to his critical illness.

## Discussion

Afib is a rapid irregular heart rhythm and the most common type of cardiac arrhythmia. Refractory cases are often treated with catheter ablation [7]. Radiofrequency energy, cryothermy, or microwaves are used to create scars in the atrial walls to block pathological, electrical pathways [4]. This procedure can inadvertently create an AEF as a complication. The esophagus lies posterior to the left atrium, which is bounded by the aorta and spine and varies in its proximity to the left atrium and pulmonary veins [1,8]. This variation leads to risks of esophageal damage during radiofrequency ablation, especially given its position behind the left atrium. The posterior left atrium wall is unevenly thick, and patients with Afib and left atrial enlargement often have increased LA-esophageal contact area and thinner fat pads [8]. This can include the esophageal vessels. Due to this anatomical structure, if esophageal necrosis occurs, it may lead to severe complications such as the formation of a fistula, which connects the esophagus to the pericardium.

Catheter ablations have been associated with esophageal complications in up to 47% of cases [1], including perforations, AEFs, and peri-esophageal nerve damage. AEFs have been reported to arise in 0.03-1% of catheter ablation cases [2-4].AEFs allow air emboli to enter the systemic circulation. Compromised blood flow can result in ischemia and infarctions to various organs [1]. AEF-related mortality rates range from 33% with surgical intervention and 96% with medical management (i.e., antibiotics) alone to 100% with just stent placement [9]. Early surgical intervention is critical for improved survival rates. Treatment includes providing supplemental oxygen, repairing the fistula, and hyperbaric oxygen therapy [9]. Both supplemental and hyperbaric oxygen therapy seem counterintuitive in patients suffering from air embolisms. However, they help dissolve the emboli while providing oxygenation to the ischemic tissues [10]. Furthermore, AEFs result when all histologic layers between the atrial and esophageal walls are affected, thus making them difficult to manage once formed [9]. Most patients develop sepsis due to the extravasation of esophageal flora into systemic circulation post-ablation [11]. Early detection and aggressive treatment are key to preventing death.

Imaging techniques like transesophageal echocardiograms and esophagoscopy may seem suitable for diagnostic workup of esophageal issues. However, both modalities can introduce more air into the systemic circulation [11,12]. If an AEF is suspected, contrast-enhanced thoracic CT or MRI should be utilized to reveal pneumomediastinum, pneumopericardium, or a direct connection between the left atrium and the esophagus [1]. Initial symptoms may include fever and polymicrobial bacteremia, which can respond quickly to antibiotics. Additionally, head CT scans without contrast can help detect pneumocephalus [4].

Pneumocephalus occurs when a fracture in the posterior skull and a dural tear enables air to enter the intracranial cavity. Tension pneumocephalus results from air accumulating under pressure in the subdural region, which potentially leads to brain compression and midline shift. Air that is trapped under pressure in the subdural region exerts a compressive effect on the brain, similar to a hematoma. This condition often arises when air enters as cerebrospinal fluid leaks out [5,6]. Patients with postoperative pneumocephalus commonly present with altered mental status and headaches. Pneumocephalus can be diagnosed with an X-ray of the skull coupled with an in-depth look via a CT or an MRI. CT is the diagnostic modality of choice for diagnosing pneumocephalus. Emergency surgical decompression is usually utilized while treatment consists of antibiotics, anti-seizure medication, and management of intracranial pressure.

## Conclusions

AEF is a rare but fatal complication of left atrial ablation therapy, a treatment for persistent Afib. It has the potential to introduce air into the systematic circulation, which can result in neurological complications. This report highlights the development of an AEF resulting from complications of left atrial ablation therapy, which further led to the diagnosis of pneumocephalus after the onset of symptoms. If AEF is swiftly diagnosed, surgical repair and acute oxygen therapy can be implemented. However, this was not the case with our patient and he passed away due to complications.
